# Supplementary material for: Parallel evolution of Pseudomonas aeruginosa phage resistance and virulence loss in response to phage treatment in vivo and in vitro
Source: eLife. 2022 Feb 21;11:e73679. doi: 10.7554/eLife.73679 (PMC8912922; doi:10.7554/eLife.73679)
Supplement: Supplementary file 1. — P-values adjusted using the Tukey method of comparing a family of three estimates. “SE” = standard error. [file elife-73679-supp1.docx]

| Contrast | Estimate | SE | z-ratio | p-value |
| --- | --- | --- | --- | --- |
| Day 2 - Day 4 | - 16.5 | 10 | -1.65 | 0.2262 |
| Day 2 - Day 7 | - 16.5 | 10 | -1.65 | 0.2264 |
| Day 4 - Day 7 | 0.000001 | 5.32 | 0 | 1 |
