# Supplementary material for: Parallel evolution of Pseudomonas aeruginosa phage resistance and virulence loss in response to phage treatment in vivo and in vitro
Source: eLife. 2022 Feb 21;11:e73679. doi: 10.7554/eLife.73679 (PMC8912922; doi:10.7554/eLife.73679)
Supplement: Supplementary file 2. [file elife-73679-supp2.docx]

| Phage resistance | Phage added | Treatment replicate | Clone number |
| --- | --- | --- | --- |
| Susceptible | Once | 6 | 1 |
| Susceptible | Once | 6 | 2 |
| Susceptible | Once | 6 | 3 |
| Susceptible | Once | 3 | 4 |
| Susceptible | Once | 3 | 5 |
| Susceptible | Once | 3 | 6 |
| Susceptible | Once | 5 | 7 |
| Susceptible | Once | 5 | 8 |
| Susceptible | Repeatedly | 6 | 9 |
| Susceptible | Repeatedly | 6 | 10 |
| Susceptible | Repeatedly | 6 | 11 |
| Susceptible | Repeatedly | 1 | 12 |
