# Supplementary material for: Parallel evolution of Pseudomonas aeruginosa phage resistance and virulence loss in response to phage treatment in vivo and in vitro
Source: eLife. 2022 Feb 21;11:e73679. doi: 10.7554/eLife.73679 (PMC8912922; doi:10.7554/eLife.73679)
Supplement: Supplementary file 3. — Outlier removed states in which treatment each outlier was identified and subsequently removed. Significant p-values in bold. [file elife-73679-supp3.docx]

| Model term | Outlier removed | Chi-sq value | P-value |
| --- | --- | --- | --- |
| Treatment * Resistance | None | 1.32 | 0.5185 |
| Treatment * Resistance | Susceptible | 0.609 | 0.7824 |
| Treatment * Resistance | Control | 1.56 | 0.459 |
| Treatment * Resistance | Both | 0.785 | 0.675 |
| Treatment | None | 2.44 | 0.295 |
| Treatment | Susceptible | 6.207 | **0.045** |
| Treatment | Control | 1.501 | 0.472 |
| Treatment | Both | 4.53 | 0.104 |
| Resistance | None | 8.28 | **0.016** |
| Resistance | Susceptible | 15.44 | **0.00044** |
| Resistance | Control | 12.48 | **0.0059** |
| Resistance | Both | 19.13 | **7.023e-05** |
