# Supplementary material for: Parallel evolution of Pseudomonas aeruginosa phage resistance and virulence loss in response to phage treatment in vivo and in vitro
Source: eLife. 2022 Feb 21;11:e73679. doi: 10.7554/eLife.73679 (PMC8912922; doi:10.7554/eLife.73679)
Supplement: Supplementary file 4. — Significant p-values highlighted in bold. [file elife-73679-supp4.docx]

| Outlier removed | Contrast | Estimate | SE | t-ratio | p-value |
| --- | --- | --- | --- | --- | --- |
| None | Resistant – Single resistant | 0.0916 | 0.210 | 0.436 | 0.901 |
| None | Resistant – Susceptible | 0.439 | 0.16 | 2.698 | **0.0289** |
| None | Single resistant - Susceptible | 0.348 | 0.2 | 1.74 | 0.207 |
| Susceptible | Resistant – Single resistant | 0.069 | 0.184 | 0.375 | 0.926 |
| Susceptible | Resistant – Susceptible | 0.597 | 0.165 | 3.618 | **0.0029** |
| Susceptible | Single resistant - Susceptible | 0.528 | 0.194 | 2.714 | **0.0282** |
| Control | Resistant – Single resistant | 0.087 | 0.193 | 0.449 | 0.8953 |
| Control | Resistant – Susceptible | 0.508 | 0.151 | 3.36 | **0.0057** |
| Control | Single resistant - Susceptible | 0.421 | 0.185 | 2.28 | 0.073 |
| Both | Resistant – Single resistant | 0.085 | 0.168 | 0.506 | 0.869 |
| Both | Resistant – Susceptible | 0.581 | 0.133 | 4.36 | **0.0004** |
| Both | Single Resistant - Susceptible | 0.496 | 0.162 | 3.067 | **0.0119** |
