# Supplementary material for: Parallel evolution of Pseudomonas aeruginosa phage resistance and virulence loss in response to phage treatment in vivo and in vitro
Source: eLife. 2022 Feb 21;11:e73679. doi: 10.7554/eLife.73679 (PMC8912922; doi:10.7554/eLife.73679)
Supplement: Transparent reporting form [file elife-73679-transrepform1.docx]

***eLife’s* transparent reporting form**

We encourage authors to provide detailed information *within their submission* to facilitate the interpretation and replication of experiments. Authors can upload supporting documentation to indicate the use of appropriate reporting guidelines for health-related research (see [EQUATOR Network](http://www.equator-network.org/%20)), life science research (see the [BioSharing Information Resource](https://biosharing.org/)), or the [ARRIVE guidelines](http://www.plosbiology.org/article/info:doi/10.1371/journal.pbio.1000412) for reporting work involving animal research. Where applicable, authors should refer to any relevant reporting standards documents in this form.

If you have any questions, please consult our Journal Policies and/or contact us: [editorial@elifesciences.org](mailto:editorial@elifesciences.org).

**Sample-size estimation**

- You should state whether an appropriate sample size was computed when the study was being designed
- You should state the statistical method of sample size computation and any required assumptions
- If no explicit power analysis was used, you should describe how you decided what sample (replicate) size (number) to use

Please outline where this information can be found within the submission (e.g., sections or figure legends), or explain why this information doesn’t apply to your submission:

Sample size estimations were not computed prior to study design. Characterisation of isolates from the patient were limited in number due to the nature of the treatment being successful (24 clones at study start, 8 at day 2 and 2 clones at day 4, no clones in following samples). We chose to analyse all clones available from the patient to maximise generalizability. See methods section: “Patient information, bacteria and phage strains and phage-bacteria evolution in vivo”.

For experiments conducted in vitro, we used a standard sample size commonly used in experimental evolution studies (n = 6 per treatment/control). 6 is commonly used as this is the minimum number usually required before needed non-parametric alternatives (e.g. Sign test). See methods section: “Experimental in vitro phage-bacteria evolution”.

**Replicates**

- You should report how often each experiment was performed
- You should include a definition of biological versus technical replication
- The data obtained should be provided and sufficient information should be provided to indicate the number of independent biological and/or technical replicates
- If you encountered any outliers, you should describe how these were handled
- Criteria for exclusion/inclusion of data should be clearly stated
- High-throughput sequence data should be uploaded before submission, with a private link for reviewers provided (these are available from both GEO and ArrayExpress)

Please outline where this information can be found within the submission (e.g., sections or figure legends), or explain why this information doesn’t apply to your submission:

How often each experiment was performed is outlined in each relevant methods section. Exclusion of outliers occurred in the growth rate and biofilm analyses and is explained in-depth in Figure S1 and in the supplementary methods (“Data cleaning of biofilm dataset”).

We have included definitions of biological replicates at the end of sections: “Patient information, bacteria and phage strains and phage-bacteria evolution in vivo” and “Experimental in vitro phage-bacteria evolution”. Technical replicates were used in growth rate and biofilm analyses and are defined in their relevant sections.

**Statistical reporting**

- Statistical analysis methods should be described and justified
- Raw data should be presented in figures whenever informative to do so (typically when N per group is less than 10)
- For each experiment, you should identify the statistical tests used, exact values of N, definitions of center, methods of multiple test correction, and dispersion and precision measures (e.g., mean, median, SD, SEM, confidence intervals; and, for the major substantive results, a measure of effect size (e.g., Pearson's r, Cohen's d)
- Report exact p-values wherever possible alongside the summary statistics and 95% confidence intervals. These should be reported for all key questions and not only when the p-value is less than 0.05.

Please outline where this information can be found within the submission (e.g., sections or figure legends), or explain why this information doesn’t apply to your submission:

For statistical analysis, see methods section “Statistical analyses”. Degrees of freedom, dispersion and precision measures and p-values are provided throughout the results section or in Table S2. Data points and precision measures are included within each figure where appropriate. Figure legends define which precision measure has been used.

(For large datasets, or papers with a very large number of statistical tests, you may upload a single table file with tests, Ns, etc., with reference to sections in the manuscript.)

**Group allocation**

- Indicate how samples were allocated into experimental groups (in the case of clinical studies, please specify allocation to treatment method); if randomization was used, please also state if restricted randomization was applied
- Indicate if masking was used during group allocation, data collection and/or data analysis

Please outline where this information can be found within the submission (e.g., sections or figure legends), or explain why this information doesn’t apply to your submission:

No group allocation was relevant with respect to the patient as this was a case study approach. In terms of the analyses of virulence, growth rate and biofilm, biological replicates (bacterial isolates) were allocated according to their treatment and resistance to phage. This is outlined in the Virulence section of the methods and the same biological replicates are used in the virulence, growth rate and biofilm experiments.

**Additional data files (“source data”)**

- We encourage you to upload relevant additional data files, such as numerical data that are represented as a graph in a figure, or as a summary table
- Where provided, these should be in the most useful format, and they can be uploaded as “Source data” files linked to a main figure or table
- Include model definition files including the full list of parameters used
- Include code used for data analysis (e.g., R, MatLab)
- Avoid stating that data files are “available upon request”

Please indicate the figures or tables for which source data files have been provided:

Data files and R script files used for all data analysis and figure presentation can be found on GitHub at: <https://github.com/mcastledine96/Parallel_evolution_phage_resistance_virulence_trade-offs_invivo_invitro>

This github link has additional information and annotated scripts to indicate how figures may be recreated.

Raw sequencing files have been archived on the European Nucleotide Archive with the project accession number PRJEB47945

https://www.ebi.ac.uk/ena/browser/view/PRJEB47945
